# Renal Cell Carcinoma in a Patient With Crossed Renal Ectopia: A Case Report

**DOI:** 10.7759/cureus.60789

**Published:** 2024-05-21

**Authors:** Leticia Helena Kaça do Carmo, Luiza Giuliani Schmitt, Francisco Ewerton de Paula Uchôa, Camila Piovesan Wiethan, Giovanni Brondani Torri

**Affiliations:** 1 Department of Medical Imaging, Hematology and Clinical Oncology, Universidade de São Paulo, Ribeirão Preto, BRA; 2 Department of Radiation Oncology, University of Texas Southwestern Medical Center, Dallas, USA; 3 Department of Medicine, Universidade Federal do Ceará, Fortaleza, BRA; 4 Department of Radiology and Diagnostic Imaging, Hospital Universitário de Santa Maria, Santa Maria, BRA

**Keywords:** case report, renal cell carcinoma (rcc), crossed-fused renal ectopia, renal ectopia, partial nephrectomy

## Abstract

Crossed fused renal ectopia (CFRE) is a rare congenital anomaly in which a kidney is located on the opposite side from where its ureter connects to the bladder, merging into the other kidney. It has been linked to other rare congenital malformations, including the VACTERL association (vertebral anomalies, anal atresia, cardiac anomalies, tracheoesophageal fistula, esophageal atresia, renal anomalies, and limb abnormalities), the MURCS association (müllerian ducts, renal, and cervicothoracic spine anomalies), increased incidence of infections, obstruction, cystic dysplasia, and urolithiasis. Although the literature has documented only a small number of cases wherein CFRE coincides with neoplasia, we present the case of a 59-year-old patient with a right ectopic kidney fused to the left one and simultaneous primary renal cell carcinoma. We aim to report and discuss this case and the treatment approach, comparing it with existing literature to enhance our understanding and management of similar occurrences, as partial nephrectomy is uncommon due to the challenging anatomy of these cases.

## Introduction

Crossed fused renal ectopia (CFRE) is a rare congenital anomaly in which the kidney is located contralateral to its ureterovesical junction and merged into the contralateral kidney [1]. The autopsy incidence of this type of abnormal renal formation is estimated at 1 per 2,000 individuals [2], more commonly in males [3]. Although this condition is associated with other congenital genitourinary and multisystem malformations and complications such as urinary tract infection and hydronephrosis [4], there are only a handful of case reports that describe patients presenting with CFRE and primary renal cell carcinoma (RCC). Here, we elucidate a case of a 59-year-old patient with a right ectopic kidney fused to the left one, suffering from RCC.

## Case presentation

A 59-year-old male presented with a one-week history of gross hematuria, dysuria, and lower back pain. He had no fever or weight loss. The physical examination was unremarkable. At the first appointment, serum creatinine was 1.46 mg/dL. The remainder of his laboratory results were normal.

The patient reported a known right kidney agenesis - an incidental finding on a prior image examination - but was unable to provide more details. He had a history of smoking equivalent to 15 pack-years and a daily alcohol intake of one beer. The patient denied illicit drug use. Medical history was also significant for hypercholesterolemia, which was treated with simvastatin. He denied prior surgical history and had no family history of cancer.

**Figure 1:**
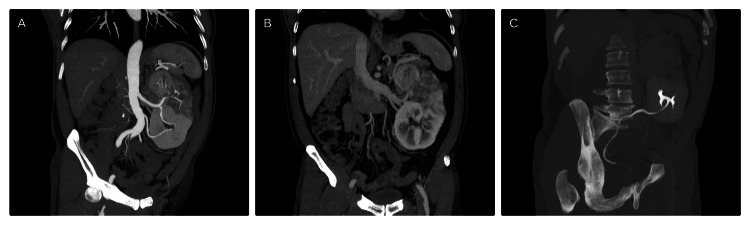
Renal cell carcinoma in crossed fused renal ectopia Coronal contrast-enhanced CT under the maximum intensity projection in arterial phase (A), corticomedullary renal phase (B), and delayed excretory renal phase (C). The arterial phase (A) shows a large unilateral kidney on the left side, with hypoenhancing mass in the upper pole. Two renal arteries are emerging from the abdominal aorta. The corticomedullary renal phase (B) shows a single draining renal vein into the IVC, with the absence of a contralateral vein. The delayed excretory renal phase (C) shows the lower moiety ureter crossing the midline, with a conventional bladder insertion. No contrast is seen within the upper moiety ureter.

Contrast-enhanced computed tomography (CT) of the abdomen and pelvis (Figure 1) revealed a single kidney on the left, with a large, heterogeneous, endophytic, expansive lesion centered in the upper pole, measuring 10.1 x 9.2 x 9.0 cm, without clear extension into the perinephric fat and collecting system. There were no signs of adjacent organ invasion. The single left kidney had two renal arteries, one single renal vein, and a duplicated collecting system, without hydronephrosis. The lower ureter crossed midline into the right vesicoureteral junction. The upper ureter had a conventional drainage into the left ureterovesical junction. The largest periaortic lymph node measured 1.1 cm in the short axis, without worrisome morphological features. No distant metastasis was detected.

A decision was made to proceed with an open partial nephrectomy, with a curative intent and complete tumor resection. After careful consideration of various factors, including the location and size of the lymph nodes, the overall clinical condition of the patient, and the potential risks associated with lymph node resection, the decision was made to not resect the lymph nodes noted on CT scan. No complications were reported, and the patient had a full, uneventful recovery.

Histology indicated a clear cell RCC (ccRCC), measuring 9.2 x 7.0 x 7.0 cm. There were no signs of angiolymphatic, perirenal capsule, or fat invasion. The tumor was staged as a pT2aNxM0. On the follow-up appointment 40 days after surgery, the patient had no symptoms or issues. He had been under surveillance with Nephrology for eight months, without any evidence of complication or tumor progression.

## Discussion

CFRE is the second most common congenital renal anomaly after horseshoe kidney, mostly affecting the left kidney [5]. The pathogenesis of CFRE involves a series of factors including the ureteric bud development, abnormal rotation, and genetic and teratogenic factors, but a definite cause of this anomaly is not known [6]. McDonald and McClellan reported in 1957 six types of CFRE: unilateral fused kidney with ectopic (kidney inferior), S-shaped kidney, lump kidney, L-shaped kidney, disc kidney, and unilateral fused kidney with ectopic (kidney superior) [7].

CFRE has been linked to other rare congenital malformations. These include vertebral anomalies, anal atresia, cardiac anomalies, tracheoesophageal fistula, esophageal atresia, renal anomalies, and limb abnormalities (also known as VACTERL association), as well as the association of müllerian ducts, renal, and cervicothoracic spine anomalies (also known as MURCS association) [8]. Normally, CFRE is asymptomatic but also associated with an increased risk of infections, obstruction, cystic dysplasia, and urolithiasis [9]. The co-occurrence of CFRE and neoplasia is extremely rare, with a few case reports describing the association and unclear conclusions regarding the increased risk of malignancy. In a recent literature review by Tsuboi et al., only 18 cases were described [10]. Most of these patients suffered from ccRCC, and only four of them underwent partial nephrectomy, with the first one occurring only in 2000 [11].

While laparoscopic or robot-assistant partial nephrectomies are preferred in cases of small masses in a regular-shaped kidney [12], treatment choices in CFRE cases are challenging due to the unique morphological features and vascular supply being variable and unpredictable [13]. This scenario can compromise early vasculature control and nephron-sparing, increasing the chances of hemorrhages, hematomas, and health-tissue ischemia [14]. Usually, there are accessory renal arteries arising from both the inferior aorta and common iliac vessels [15], but in the presenting case, the arterial supply of each moiety was derived from the aorta through renal arteries, decreasing the risk of vascular injury during the surgical procedure and dissection. Therefore, careful planning for surgery is required with contrast-enhanced imaging to maximize the preservation of non-affected moiety, which was obtained in most of the patients planned for partial nephrectomy including the patient in this case report.

In our case, the subtype of CFRE created by McDonald and McClellan was the unilateral fused kidney, as mentioned earlier. Noteworthy, the distinguishing characteristic of this case is the right-to-left side of the anomaly, making it three times less common than the other one. This study is the first to describe this condition in South America. Considering this discussion, the data available preclude any correlation between the ccRCC and the CFRE because of the small studies, mainly in emerging countries.

There are no specific recommendations for the follow-up of ccRCC patients with concomitant kidney malformations. For the general population, the need for periodic imaging is guided by factors such as the risk of cancer recurrence, patient performance status, and patient preferences [16,17]. However, evidence on the benefits of long-term postoperative imaging remains conflicting [18].

## Conclusions

In this case report, we present a rare case of RCC in a patient with right-to-left CFRE. CFRE is the second most common renal congenital abnormality, following horseshoe kidney, but remains rare. Despite the complex anatomy presented by this renal malformation, the patient successfully underwent an open partial nephrectomy with curative intent. There are no specific recommendations for the follow-up of RCC patients with concomitant kidney malformations, and the evidence on the benefits of long-term postoperative imaging remains conflicting. Given the limited number of reported cases of crossed renal ectopia associated with kidney malignancies, it is currently not possible to determine if this congenital anomaly increases cancer risk.

## References

[1] Solanki S, Bhatnagar V, Gupta AK, Kumar R (2013). Crossed fused renal ectopia: challenges in diagnosis and management. J Indian Assoc Pediatr Surg.

[2] Akdogan L, Oguz AK, Ergun T, Ergun I (2015). The rarest of the rare: crossed fused renal ectopia of the superior ectopia type. Case Rep Nephrol.

[3] Sharma VB, Babu CSR, Gupta OP (2014). Crossed fused renal ectopia multidetector computed tomography study. Int J Anat Res.

[4] Mudoni A, Caccetta F, Caroppo M (2017). Crossed fused renal ectopia: case report and review of the literature. J Ultrasound.

[5] Partin AW, Dmochowski R, Kavoussi L (2020). Campbell Walsh Wein Urology. 12th Edition. https://www.us.elsevierhealth.com/campbell-walsh-wein-urology-9780323546423.html.

[6] Babu CSR, Sharma V, Gupta OP (2015). Renal fusion anomalies: a review of surgical anatomy. Anat Physiol.

[7] McDonald JH, McClellan DS (1957). Crossed renal ectopia. Am J Surg.

[8] Foster BR, Fananapazir G (2022). Diagnostic Imaging: Genitourinary. 4th Edition. https://www.sciencedirect.com/book/9780323377089/diagnostic-imaging-genitourinary#book-description.

[9] Romero FR, Chan DY, Muntener M, Bagga HS, Brito FA, Kavoussi LR (2007). Laparoscopic heminephrectomy for renal cell carcinoma in cross-fused ectopic kidney. Urology.

[10] Tsuboi I, Ogawa K, Yokoyama S, Araki A, Kadota K, Wada K (2022). Open partial nephrectomy of a left-to-right crossed fused renal ectopia with clear cell renal cell carcinoma: case report and review of the literature. Urol Case Rep.

[11] Sugita S, Kawashima H, Nakatani T, Yoshimura R, Wada S, Sugimura K, Kishimoto T (2000). Renal cell carcinoma in an L-shaped kidney. Int J Urol.

[12] Krabbe LM, Bagrodia A, Margulis V, Wood CG (2014). Surgical management of renal cell carcinoma. Semin Intervent Radiol.

[13] Boyan N, Kubat H, Uzum A (2007). Crossed renal ectopia with fusion: report of two patients. Clin Anat.

[14] Stimac G, Dimanovski J, Ruzic B, Spajic B, Kraus O (2004). Tumors in kidney fusion anomalies--report of five cases and review of the literature. Scand J Urol Nephrol.

[15] Türkvatan A, Ölçer T, Cumhur T, Akdur PO (2008). Multidetector computed tomographic urography for evaluation of crossed fused renal ectopia. Ankara Üniversitesi Týp Fakültesi Mecmuasy.

[16] Motzer RJ, Jonasch E, Agarwal N (2022). Kidney Cancer, Version 3.2022, NCCN Clinical Practice Guidelines in Oncology. J Natl Compr Canc Netw.

[17] Campbell SC, Uzzo RG, Karam JA (2024). Renal Mass and Localized Renal Cancer: Evaluation, Management, and Follow-up: AUA Guideline: Part II. J Urol.

[18] Dabestani S, Beisland C, Stewart GD (2019). Increased use of cross-sectional imaging for follow-up does not improve post-recurrence survival of surgically treated initially localized R.C.C.: results from a European multicenter database (R.E.C.U.R.). Scand J Urol.

